# Estrogens as a Possible Therapeutic Strategy for the Management of Neuroinflammation and Neuroprotection in COVID-19

**DOI:** 10.2174/1570159X21666230616103850

**Published:** 2023-08-15

**Authors:** Cindy Bandala, Noemí Cárdenas-Rodríguez, Samuel Reyes-Long, Alfredo Cortés-Algara, Itzel Jatziri Contreras-García, Teresita Rocío Cruz-Hernández, Alfonso Alfaro-Rodriguez, José Luis Cortes-Altamirano, Martín Perez-Santos, Maricruz Anaya-Ruiz, Eleazar Lara-Padilla

**Affiliations:** 1 Higher School of Medicine, National Polytechnic Institute, Mexico City, 11340, Mexico;; 2 Neuroscience Laboratory, National Institute of Pediatrics, Mexico City, 04530, Mexico;; 3 Basic Neurosciences, National Institute of Rehabilitation LGII, Mexico City, 14389, Mexico;; 4 Department of Robotic Surgery and Laparoscopy in Gynecology, Centro Médico Nacional 20 de Noviembre, Mexico City, CP, Mexico;; 5 Research Department, Ecatepec Valley State University, Valle de Anahuac, Ecatepec, 55210, Mexico State, Mexico;; 6 Directorate of Innovation and Knowledge Transfer, Meritorious Autonomous University of Puebla, 72570, Puebla;; 7 Cell Biology Laboratory, Oriente Biomedical Research Center, Mexican Social Security Institute, Metepec, 74360, Puebla

**Keywords:** Estrogens, ACE2, Ang (1-7), SARS-CoV-2, COVID-19, neuroprotection, neuroinflammation

## Abstract

The Coronavirus disease 2019 (COVID-19) affects several tissues, including the central and peripheral nervous system. It has also been related to signs and symptoms that suggest neuroinflammation with possible effects in the short, medium, and long term. Estrogens could have a positive impact on the management of the disease, not only due to its already known immunomodulator effect, but also activating other pathways that may be important in the pathophysiology of COVID-19, such as the regulation of the virus receptor and its metabolites. In addition, they can have a positive effect on neuroinflammation secondary to pathologies other than COVID-19. The aim of this study is to analyze the molecular mechanisms that link estrogens with their possible therapeutic effect for neuroinflammation related to COVID-19. Advanced searches were performed in scientific databases as PubMed, ProQuest, EBSCO, the Science Citation index, and clinical trials. Estrogens have been shown to participate in the immune modulation of the response to severe acute respiratory syndrome coronavirus 2 (SARS-CoV-2). In addition to this mechanism, we propose that estrogens can regulate the expression and activity of the Angiotensin-converting enzyme 2 (ACE2), reestablishing its cytoprotective function, which may be limited by its interaction with SARS-CoV-2. In this proposal, estrogens and estrogenic compounds could increase the synthesis of Angiotensin-(1-7) (Ang-(1-7)) that acts through the Mas receptor (MasR) in cells that are being attacked by the virus. Estrogens can be a promising, accessible, and low-cost treatment for neuroprotection and neuroinflammation in patients with COVID-19, due to its direct immunomodulatory capacity in decreasing cytokine storm and increasing cytoprotective capacity of the axis ACE2/Ang (1-7)/MasR.

## 1 INTRODUCTION

Coronavirus disease 2019 (COVID-19) has shown a pandemic behavior with a wide spectrum of clinical manifestations. This disease is caused by SARS-CoV-2, a Betacoronaviridae member, as other viruses are associated with severe acute respiratory syndromes (SARS-CoV and MERS) [1]. SARS-CoV-2 is a single-stranded positive-sense RNA virus, containing approximately 26-32 kb genome. The viral structure consists of an envelope bilayer lipidic membrane with several structural proteins, including the spike protein. Also, some variants have been identified contributing to viral genome heterogeneity [2]. Angiotensin-converting enzyme 2 (ACE2) is the viral receptor, and its gene and protein expression have been identified in different tissues, such as the nervous system, which allows viral tropism and infection. COVID-19 shows multiple symptoms, however, the main ones are fever, cough, fatigue, muscle aches and anorexia, and one of the most important complications is that COVID-19 has been related to neuroinflammation in both the central and peripheral nervous systems [3]. Currently, COVID-19 has no effective treatment, nevertheless, has been registered to develop potential therapeutic agents such as anti-inflammatory drugs, immunomodulators, anti-vascular endothelial growth factors, modulating drugs, or nutritional supplements [4]. Neuroprotection and treatment of neuroinflammation are two important drug targets in the COVID-19 patients, in order to prevent future neurodegenerative diseases and improve symptoms associated. In this sense, estrogens could be an option because it has been shown, that they cross the blood-brain barrier efficiently and could have an immunomodulatory effect on nervous tissue because the cells have receptors to provoke these mechanisms of action [5]. Clinical studies have shown that estrogen derivatives are effective and safe in reducing respiratory complications in patients with COVID-19. However, their effect on the possible neuroinflammation that could accompany this disease has not yet been demonstrated.

## 2 METHODOLOGY

Advanced searches were performed in scientific databases such as PubMed, ProQuest, EBSCO, Scopus, Science Direct, Clarivate, Google Scholar, World Health Organization (WHO) and Clinical Trials. The original manuscripts were selected using the following keywords alone or in combination: “estrogens”, “neuroinflammation”, “COVID-19”, “Brain ACE2”, “Brain Angiotensin 1-7”, “gene expression”, “protein expression”, “epidemiology”, “Symptoms”, “Signs”, “Renin-Angiotensin System”, “Central Nervous System”, “Peripheral Nervous System”. To obtain the patent documents relating to neuroinflammation, a search strategy based on keywords related to neuroinflammation, neuroprotective and estrogen was designed. This strategy included all patents published until December 2021 in the patent databases of the world's main patent offices, United States Patent and Trademark Office, European Patent Office, World Intellectual Property Organization, Japan Patent Office, China State Intellectual Property Office, and Korean Intellectual Property Office. The patent search was performed using keywords in the title, abstract and claims sections of the patent databases. After neuroinflammation/estrogen patents were obtained, they were evaluated to avoid duplication and ensure that they were relative to the topic. A total of 118 references were included, ranging from the year 2004 to 2022.

## 3 EPIDEMIOLOGICAL CRITERIA OF SEVERE COVID-19 RELATED TO THE SEX, AGE AND COMORBIDITIES

**Fig. (1):**
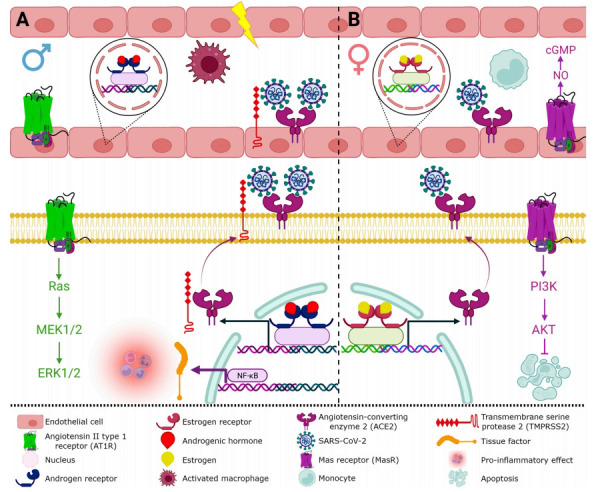
Potential sex differences are involved in the severity and the less favorable outcome of COVID-19. (**A**) SARS-CoV-2 entry into the cell requires the attachment to the angiotensin-converting enzyme 2 (ACE2) and the transmembrane protease serine 2 (TMPRSS2) facilitation membrane fusion and entry. Activation of androgen receptors increases the expression of TMPRSS2 and ACE2 by promoting their transcription, however, the internalization of ACE2 with SARS-CoV-2 diminishes the conversion of Ang-II to Ang-(1-7). In turn, Ang-II is available to bind with AT1R to generate inflammation, vasoconstriction and fibrosis. Thus, androgens are immunosuppressive factors that generate rapid spread of SARS-CoV-2. (**B**) Activation of estrogen receptors downregulates the expression of AT1R and promotes the expression of ACE2, AT2R, and MasR, without increasing the synthesis of TMPRSS2, resulting in anti-inflammatory and vasodilatation effects. Also, estrogens have an immunostimulatory function by increasing the release of pro-inflammatory cytokines, and, although it seems paradoxical, the sex differential expression in the innate cell allows better control of the entry of the SARS-CoV-2 to the cell.

Several epidemiological reports around the world have shown that women are less susceptible to SARS-CoV-2 infection and are less likely to have severe symptoms and mortality [5]. Other studies have shown that older patients with diabetes, hypertension and metabolic syndrome have higher mortality [6]. In line with this, Shi *et al.* reported that in 487 COVID-19 Chinese patients, the most affected had characteristics like elder age (OR 1.06 (95% CI 1.03-1.08), *p* < 0.001), or male gender (OR 3.68 (95% CI 1.75-7.75), *p* = 0.001). The most frequent comorbidities reported were hypertension (53.1%) and Diabetes mellitus type 2 (DM2) (14.3%) [7]. Plus, Qin *et al.* showed in 548 COVID-19 patients that males had higher mortality than females (22.2% *vs*. 10.4%) and the same fact was observed in older age. A possible explanation of this effect is the exacerbated inflammatory response that men show; higher levels of Tumor Necrosis Factor α (TNF-α), lactate dehydrogenase (LDH), ferritin and high-sensitivity C-reactive protein (hsCRP), but lower lymphocyte count than females, adjusted by age and comorbidity (Fig. **1A** and **B**) [8]. ACE2 was identified as the receptor for SARS-CoV-2 [9]. Chen *et al.* described a higher ACE2 expression in Asian females that significantly decreased during aging in many tissues and found a strong negative correlation of ACE2 expression with viral infection. Also, they showed that ACE2 expression increases in adulthood at the lungs, brain, and kidneys [10]; this is an important factor that could help us explain the highest viral tropism in relation to the age group with the highest infection and complication rates, but this fact is not yet completely clear. DM2 has been related to severe COVID-19 and mortality, plus, it has been observed that DM2 could decrease ACE2 and estrogens levels.

## 4 SYMPTOMS, SIGNS AND COMPLICATIONS IN THE CENTRAL NERVOUS SYSTEM CONSEQUENCE OF COVID-19

In a case series in Wuhan, China, 36.4% of COVID-19 cases experienced central nervous system (CNS) symptoms, 8.9% peripheral nerve system (PNS) symptoms, and 10.7% reported skeletal muscle symptoms [11]. The main CNS symptoms reported in COVID 19 at the beginning of the pandemic were dizziness (16.8%) and headache (13.1%). Other CNS complications were impaired consciousness, acute ischemic stroke and intracranial hemorrhage. Additionally, the main PNS symptoms were hypogeusia (5.6%) and hyposmia (5.1%) [12]. However, over time, new and more complex neuronal complications have been reported (Table **1**). A meta-analysis carried out by Ahmed *et al.* in 2022 reports the frequency of appearance of neuronal complications, with Guillain-Barré syndrome appearing more frequently, secondly stroke and subsequently, optic neuritis, encephalitis, myopathy, transverse myelitis, critical illness neuromyopathy/neuropathy, encephalopathy, status epilepticus, Bell’s palsy, parkinsonism, vestibulocochlear neuritis and lastly opsoclonus myoclonus syndrome [13]. On the other hand, there is an interesting report of a COVID-19 patient with rare symptoms; the primary manifestation leading to admission was third nerve palsy; however, on the second hospital stay day, the features progressively evolved with fever and dyspnea [14]. Studies on SARS-CoV and MERS revealed that a minority of patients do not return to normal life quality after infection and may experience several neurological complications even for years after acute infection [15].

## 5 THE ACE2/ANG-(1-7) /MASR AXIS IN NEUROINFLAMMATION

**Fig. (2):**
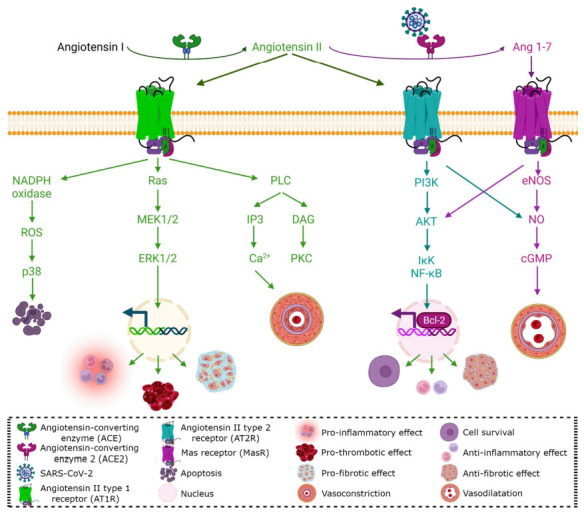
ACE/AngII/ATR1 and ACE2/ Ang-(1-7)/MasR axis. Angiotensin I (Ang I) is catalyzed to angiotensin II (Ang II) by the angiotensin-converting enzyme (ACE); then, Ang II is converted to Ang-(1-7) by the action of angiotensin-converting enzyme 2 (ACE2), which is the SARS-CoV-2 receptor. Ang II interacts with its two receptors ATR1 and ATR2. Binding of Ang II to ATR1 activates Ras and PLC pathways, as well as NADPH oxidase, resulting in pro-inflammatory, pro-thrombotic, pro-fibrotic, pro-oxidative effects, vasoconstriction, and apoptosis. While ATR2 and MasR activation leads to PI3K and an increase of eNOS, generating a decrease in tissue damage due to an anti-inflammatory, anti-thrombotic, anti-oxidative, and vasodilatation effect.

It has been demonstrated that estrogens could regulate immunological response to different viral diseases, Particularly, a study in an animal model of hypertension has shown that estrogens could increase Angiotensin-(1-7) (Ang-(1-7)) expression in different tissues, showing the possible function of estrogens in COVID-19 [10]. ACE2 is a carboxypeptidase involved in the cleavage of angiotensin I and angiotensin II in human cells (Fig. **2**) [19]. Similar to other coronaviruses, during the viral entry, the spike proteins on the envelope of SARS-CoV-2 are cleaved into S1 and S2 subunits being the S1 protein/receptor determinant for the infection in the host species. S1 contains the receptor binding domain and binds to the peptidase domain of ACE2 to enter the cell [20]. Cleavage of S1 is mainly dependent of Transmembrane Serine Protease 2 (TMPRSS2) [21]. The binding of S1 to ACE2 receptor cleavages ACE2 by the action of A Disintegrin and Metalloproteinase 17 (ADAM17). This, plus TMPRSS2 cleavage, facilitate viral entry [22]. As expected, after unveiling the viral receptor, clinical studies were performed testing ACE2 inhibitors, which are available for the management of hypertension. Surprisingly, the results showed that patients did not improve. Inhibition of ACE2 has been postulated to increase its expression by negative feedback, but this mechanism has not been clearly demonstrated [23]. ACE regulates the renin-angiotensin system (RAS). The RAS, and its main product Ang-(1-7), is the major regulator of the pathophysiological mechanisms of COVID-19. The RAS imbalance causes the activation of ACE/AngII/AT1R, causing a hyperinflammatory state and pulmonary injury, thus, inducing various signaling pathways, including ERK, JNK/MAPK as well as PKC and suppressing the anti-inflammatory alternative pathway ACE2/Ang-(1-7)/MasR being this the ACE2 paradoxical action in COVID-19 (Fig. **2**) [24, 25]. Ang-(1-7), which upon binding to MasR is able to reduce the lung injury throughout a decrease in cytokine storm and oxidative stress with the consequent anti-inflammatory, antioxidative and anti-fibrotic effects [26]. SARS-CoV-2 binding to ACE2 impairs the possible cytoprotective capability of ACE2/Ang-(1-7)/MasR not only in the lungs, but also in other tissues. It has been postulated that the exogenous human recombinant ACE2 could be an alternative to COVID-19 treatment [27]. Supporting this, pharmaceutical preparations of recombinant ACE2 administered in animal models protect against lung cell death, inhibiting acute lung injury and preventing fibrosis after chronic damage to the lungs [28]. For these reasons, the activation of RAS through the ACE2/Ang-(1-7)/MasR axis can be considered for treatment of COVID-19 as has been previously proposed [29].

## 6 SEXUAL HORMONES AND COVID-19: THE RELATION BETWEEN ESTROGENS AND ACE2/ANG-(1-7)/MASR AXIS IN THE BRAIN

**Fig. (3):**
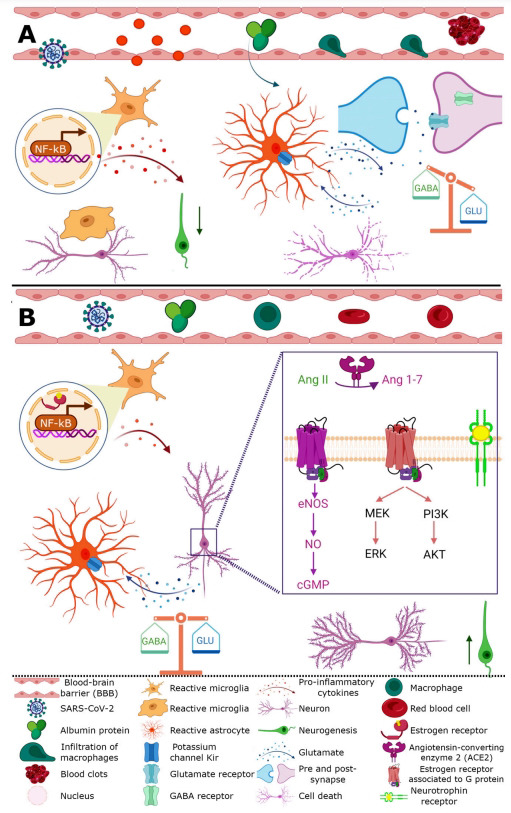
(**A**) SARS-CoV-2 could enter to brain parenchyma across the blood-brain barrier (BBB). The high levels of pro-inflammatory cytokines that circulate in the bloodstream could break the BBB, allowing infiltration of macrophages, proteins, and more cytokines. All these factors can generate a switch in the phenotype of glial cells (astrocytes and microglia), that increase the synthesis of pro-inflammatory interleukins, thereby decreasing neurogenesis. Microglial activation has been associated with cognitive impairment, probably due to decreased dendritic spines and/or synaptic contacts. In COVID-19 has been reported the triggering of seizures may be for a disbalance between excitatory and inhibitory transmitters. Reactive astrocyte increases the release of glutamate and decreases its recapture, augmenting neuronal excitability; in addition to increasing the GABA receptor internalization. The increase of cytokines and excitatory neurotransmitters provokes cell death. The binding between SARS-CoV-2 and ACE2, increases angiotensin II, activating to AT1 receptor and exerting pro-inflammatory and vasoconstrictor effects. (**B**) It has been suggested that estrogens are neuroprotective molecules that could be used in the treatment against COVID-19. Estradiol promotes, by genomic mechanism, ACE2 increase, which allows catalyzing angiotensin II to Ang-(1-7), subsequently, the latter binding to AT2 and Mas receptors activating PI3K and eNOS pathways resulting in vasodilatation, anti-inflammatory, anti-oxidative, and balance among glutamatergic and GABAergic systems. Additionally, estrogen receptors accoupled to G protein activate PI3K and ERK pathways and thereby increasing cell survival, also promote the synthesis of neurotrophic factors which in turn increases neurogenesis and neuronal plasticity.

A recent meta-analysis of 145,721 cases of COVID-19 indicated that SARS-CoV-2 could cause damage to the CNS and PNS, reporting fatigue, myalgia, headache, seizure, ataxia, cognitive impairment, stroke, among others [30, 31]; some of these symptoms have been related with factors such as hypoxia and the cytokine storm acting together with neurotrophic effects of SARS-CoV-2 [31]. It has been proposed that the increase in circulation of peripheral pro-inflammatory cytokines could lead to a breakdown of the blood-brain barrier (BBB), which would then allow SARS-CoV-2 to enter the brain parenchyma causing glial reactivity and, with it, neuroinflammation, neurodegeneration, and ultimately death cell (Fig. **3A**) [32]. It is known that female sexual hormones may modulate the inflammatory response and affect the immune system [33], however, the relation between the effect of estrogens (17β-estradiol), androgens (testosterone) and COVID-19 progression is not clear yet. As previously mentioned, sex is an important factor involved in the severity and mortality of SARS-CoV-2, reporting that both are higher in men than women, indicating that estrogens could have a protective effect while androgens do not [34]. Epidemiological studies have shown that in countries such as China, Germany and Italy strong associations exist between serum testosterone levels, inflammatory cytokines, disease progression and clinical outcomes in male with COVID-19, independent of age and comorbidities [35]. Besides, it has been shown that high levels of testosterone in serum can increase expression of ACE2 resulting in thrombotic risk, immune system and endothelial dysfunction and systemic inflammation affecting the prognosis in men with COVID-19 [36]. In contrast, a study of network analysis reported that estrogen treatment decreases mortality associated with COVID-19, where high levels of estradiol present in young females are protective by acting on nuclear receptors decreasing inflammatory response associated with SARS-CoV-2 [34].

Previous studies indicated that the fluctuation of estrogens levels in the menstrual cycle can induce immunoreactive differences in women; in the ovulation estrogen levels are high. Similarly, this protection remains during the treatment with hormonal contraception replacement or treatment with postmenopausal hormone replacement therapy [5]. In the past, it has been shown that pregnancy and lactation induced a marked adaptation of the hypothalamus-pituitary adrenal (HPA) axis, changes in behavioral and neuroendocrinal alterations together with dramatic hormonal variations that result in a decrease in stress, inflammatory response and in excitotoxic, damage in the brain of the mother [37-40]. In relation to hormones, fluctuation during pregnancy and neuroinflammation has been shown to affect the woman's immunological state [41]. In neurological diseases, it has been shown that pregnancy affects women with multiple sclerosis strongly affect the disease activity and is related with progesterone and estrogen fluctuations observing reduction in relapse rates [42-43]. Rossi and coworkers in 2018, through mass spectrometry analysis, showed that 12 pregnant women were affected with multiple sclerosis fluctuations in estrogen and progesterone during the three trimester and the post-partum period and showing changes with an increase in other metabolites such as steroids, amino acids and sphingolipids. The authors observed an increase in estrogens and progesterone being correlated with maternal immunomodulation and neuroprotection together with other metabolites that were segregated in the pregnancy in comparison with non-pregnant women. The authors concluded that neuroprotection in pregnancy is a complex mosaic of changes in sexual hormones and in other metabolites [44]. Changes in inflammatory parameters also were observed in pregnant women. In a retrospective study with 30 Chinese pregnant and 42 non-pregnant women with COVID-19 was observed that in pregnancy, the women showed milder symptoms with a high rate of asymptomatic infection, shorter length of hospital stay but was observed an increase in inflammatory markers such as white blood cell count, neutrophil count and percentage, C-reactive protein, procalcitonin, and D-dimer in comparison with non-pregnant [45]. Other studies have also found pregnant COVID-19 patients with similar clinical characteristics and disease outcomes as non-pregnant women [46, 47]. Other reports observed higher rates of admission to ICU and a higher risk for more severe COVID-19 cases among pregnant compared with non-pregnant patients [48-50]. These studies considered that the presence of advanced maternal age, and comorbidities such as diabetes mellitus, hypertension and obesity could be possible risk factors for severe COVID-19 development in pregnant women [51]. However, pregnancy in the COVID-19 occurrence needs further research. In relation to sex hormones and longer duration of COVID-19, research suggests that women present a greater proportion in comparison with men. Some reasons for this phenomenon can be socio-economic conditions, mental health, psycho-physiological factors and age under 50 years; all of these are now considered risk factors for this condition. Moreover, many symptoms of long COVID-19 as fatigue, muscle aches, palpitations, cognitive impairment and sleep disturbance have an overlap with perimenopause and menopause and affect women of all ages, prompting hormone replacement therapy as a promising option for its treatment and also for reduction of other symptoms that increase at these stages such as cardiovascular diseases, diabetes, osteoporosis, dementia, memory problems, brain fog, fatigue, anxiety, low mood, join paints and headaches. Some of these conditions are like COVID-19 and are present in both perimenopause and menopause in women, which would help us discriminate the diagnosis of long COVID-19 and to identify the molecular mechanisms of this pathology in both sexes [52-57]. The role of mast cell activation syndrome (MCAS) has been explored in long-COVID-19 women, due to its hyperinflammatory behavior and its role in the procoagulant state influenced by estradiol and progesterone. Also, it has been proposed that the presence of autoimmune pathogeneses exacerbates the condition of long COVID-19 in women [53, 58, 59]. Based on the information displayed above, we propose that there could be a positive correlation between estrogens and neuroprotection in patients infected with SARS-CoV-2 with neurological symptoms.

Estrogens are steroid sex hormones that act on two types of receptors and exert genomic and nongenomic mechanisms: classical receptors that are nuclear ligand-gated transcription factors (estrogen receptors, ERα and ERβ) and receptors associated with G proteins localized in the cell membrane which stimulate several pathways including extracellular signal-regulated kinase (ERK) and phosphatidylinositide 3-kinase (PI3K) [60-62].

At the peripheral level, estrogens act differentially in immune cells, ERα is highly expressed on T cells and ERβ on B cells stimulating the humoral response to viral infections inducing high levels of antibodies [5]. At central level, neurons, endothelial cells, glial cells, microglia, and astrocytes, have receptors for estradiol, with a higher expression of ERα in glial cells [61, 63]. It should be noted that all types of estrogens, estriol, estrone, and estradiol, have neuroprotective effects through the activation and inhibition of several pathways, among them RAS [61, 64]. The binding of Ang II and AT1 receptor increases blood pressure by inducing vasoconstriction, while the overexpression of ACE2 attenuated hypertension through the upregulation of the expression and phosphorylation of nitric oxide synthase (NOS), increasing, in turn, nitric oxide (NO) release (Fig. **2**) [65]. The activation of the proinflammatory component of the RAS pathway induces vasoconstriction, and increases pro-inflammatory cytokines, principally interleukin-6 (IL-6), which activate the coagulation cascade, and therefore, increase the risk of thrombosis (Fig. **3A**) [32, 66]. This has been observed in brain autopsies of individuals infected with SARS-CoV-2 as one of the main events associated with brain damage [67, 68]. Even though concrete evidence is still missing on the relation between sex hormones and ACE2/Ang-(1-7)/MasR axis with COVID-19 progression, the treatment with estrogens could have vasculo-protective and neuroprotective effects by reducing signs of vascular impairment, reducing platelet aggregation, modulating molecular pathways on endothelial cells, regulating vascular tone, and reducing thrombotic risk and inflammatory process (Fig. **3B**) [41, 45, 46, 64, 68, 69]. Interestingly, it has been reported that the androgens enhance platelet aggregation [45, 68], which could explain the increased risk of neurovascular damage in men with respect to women (Fig. **1**). The neuroprotective effect observed in women could be due to genomic and non-genomic estrogen effects; estrogen inhibits ACE enzyme, reducing angiotensin II and thereby inhibiting the activation of the AT1 receptor; in addition, it also promotes the synthesis and enzymatic activity of ACE2, stimulating the protective RAS pathway through activation of AT2 and Mas receptors resulting in an increase in NO, an endothelial-derived mediator involved in vasodilation and therefore in inhibition of thrombosis (Fig. **3B**) [41, 45, 46, 64, 68, 69]. This is supported by Hilliard *et al.* who showed evidence that 17β-estradiol can modulate the RAS by increasing the ACE2/Ang-(1-7)/MasR expression, plus they showed that this system was upregulated in premenopausal females and can protect against hypertension, renal and cardiovascular diseases in comparison to men. Recently, it has been suggested that 17β-estradiol can be used as an antiviral therapy against SARS-CoV-2 stimulating the generation of NO and attenuating the vasoconstrictor response [70].

On the other hand, it has also been reported that the SARS-CoV-2 breaks the BBB, allowing infiltration of macrophages, peripheral cytokines, and proteins, such as albumin, generating the activation of microglia and astrocytes that facilitates the release of pro-inflammatory cytokines, which in turn increase reactive oxygen species (ROS) production, resulting in the trigger of seizures (Fig. **3A**) [32, 61]. The change in the microglial phenotype was observed in patients and animal models with COVID-19 [67, 71]. In studies conducted on postmortem of individuals infected with SARS-CoV-2, a prominent microglial activation was observed, however, inflammation was minimal probably by means of immunosuppressive therapy [67]. In the same way, macaques infected with SARS-CoV-2 showed infiltration of T-cells and activated microglia in the brain parenchyma even in the absence of viral antigen [71]. Estrogens exert anti-inflammatory effects on innate immune responses by reducing monocyte and macrophage inflammatory cytokine release, delaying neutrophil apoptosis, and enhancing neutrophil annexin-1 expression without increasing their activation [72, 73]. It has been shown that estrogens have an activity against respiratory viral infection in female mice infected with H1N1 influenza [74]. Even though the association between neuroinflammation, gender, and COVID-19 has not been reported, *in vitro* studies show that microglia have a higher phagocytic function in females *vs*. males, plus, males express a greater quantity of pro-inflammatory genes while females express more genes associated to cellular repair [61]. As mentioned above, estradiol regulates immune response in the brain; in microglial culture, the binding estradiol-ERα inhibits the nuclear factor NF-κB preventing inflammatory gene transcription. In these conditions, the effect of estradiol on their receptor was through nongenomic signaling when PI3K pathway is activated (Fig. **3B**) [75]. In this manner, in two different studies, in a longitudinal study and a post-mortem case series study, astroglia activation was detected in patients with COVID-19 [76-78]. This activation could increase pro-inflammatory cytokines levels differentially among men and women, which is supported by the fact that in cortical astrocyte cultures, from male or female mice incubated with lipopolysaccharide (LPS), higher levels of pro-inflammatory cytokines IL-6, TNFα, and IL-1β were observed in astrocytes derived from males, which in turn could be related to testosterone levels [79].

A protective effect of estradiol on astrocytes has also been observed. In mice with allergic encephalomyelitis, a chronic neuroinflammation model, estrogen treatment decreases astrocytic C-C motif chemokine ligand 2 (CCL2); this anti-inflammatory action was reproduced in astrocyte cultures. The anti-inflammatory pathway is mediated by the suppression of NF-κB-dependent transcriptional activity, decreasing the release of inflammatory mediators and triggering anti-inflammatory action (Fig. **3B**) [61, 63]. Likewise, the activation of nuclear estrogen receptors decreases ROS levels and modulates NADPH oxidase and AT1 receptor gene expression (Fig. **3B**) [64, 80]. Additionally, Chen *et al.* established a negative correlation between estrogen and androgen hormones with ACE2 expression and COVID-19 patient’s mortality. In this study, using GTEx gene expression data and analysis, it was observed that in Asian females, young people and in type 2 diabetes patients, the ACE2 expression is high whereas its decrease is age-dependent in all ethnic groups. Moreover, ACE2 expression is reduced in DM2 patients with inflammatory cytokine treatment but upregulated by estrogen and androgen (both decrease with age) [10]. Stelzig *et al.* [81] showed that 17β-estradiol downregulates the gene expression of ACE2, and it does not affect the TMPRSS2 gene expression in differentiated normal human bronchial epithelial cells from a female donor [59, 81]. In the same way, estrogen showed a neuroprotective effect in a model of cerebral ischemia and in an Alzheimer’s disease model. Ovariectomized rats showed higher brain damage after ischemia was induced by middle cerebral artery occlusion compared to control rats, while ovariectomized rats were pretreated with olmesartan and showed an increase in the activation of ERα, which decreased brain damage through an ACE2 upregulation and reduction angiotensin II (Ang II) [82]. Meanwhile, ovariectomized rats showed an increase in inflammatory biomarkers (TNF-α and IL-1β) and cognitive impairment with over-activation of ACE/Ang II/AT1R axis in hippocampus; however, the administration of telmisartan or 17β-estradiol caused a neuroprotective effect with the expression of ACE2/Ang-(1-7)/MasR axis [83]. Another neurological symptom present in patients with COVID-19 is the appearance of epileptic seizures [31] due to an imbalance between excitatory and inhibitory neurotransmitters associated with inflammatory cytokines (Fig. **3A**) [32]. In physiological conditions, astrocytes participate in glutamatergic synaptic function controlling the amount of neurotransmitters present in cleft synaptic. However, under inflammatory conditions, an increase in the release of glutamate from astrocytes has been observed, plus, a decrease in the reuptake of this excitatory neurotransmitter from glial cells by downregulation of potassium channels Kir, resulting in higher potassium and glutamate concentration in the synaptic cleft due of bind between albumin and transforming growth factor-beta (TGF-β) expressed in this cell [32]. Additionally, inflammatory cytokines promote the internalization of gamma-aminobutyric acid (GABA) receptors, and increase the entry of calcium into neurons through N-methyl-D-aspartate (NMDA) receptors. All these factors trigger neuronal hyperexcitability, seizures, and epilepsy (Fig. **3A**) [32]. Finally, the increase of pro-inflammatory cytokines, the massive release of glutamate, and the increase of calcium into the cell induce apoptosis mainly in the hippocampus (Fig. **3A**) [32]. Estradiol promotes neuronal survival by increasing its expression in neurons and glial cells, generating neuroprotective effects, and regulating inflammatory and vascular responses on glial and endothelial cells through activation of PI3K/AKT. Also, estradiol increases brain-derived neurotrophic factor (BDNF) and its receptor expression, resulting in the activation of protective pathways generating upregulation of antiapoptotic and downregulation of proapoptotic and proinflammatory genes. In turn, estrogen increases antioxidant enzyme expression, including manganese superoxide dismutase (Mn-SOD) and glutathione synthase (Fig. **3B**) [62]. Based on these studies, estradiol could be a promising candidate for patients with COVID-19 by reducing neuroinflammatory processes and generating neuroprotection. Finally, it has been reported that active microglia and astrocytes increase IL-6 production, a cytokine involved in diminishing long-term potentiation in the hippocampus, while estradiol enhances the expression of BDNF, a molecule that regulates synaptic transmission and activity-dependent plasticity (Figs. **3A** and **3B**) [32, 84]. Shete has proposed the urgent need to evaluate the use of agonists of as treatment for patients with COVID-19 by its anti-hypertensive, antiarrhythmic, anti-inflammatory, antioxidant, and anti-fibrotic effects [85]. Also, Malek Mahdavi has proposed the use of vitamin D in patients with COVID-19 by acting as a negative-endocrine RAS modulator and inhibits renin expression and induce ACE2/Ang-(1-7)/MasR axis activity [86].

## 7 ESTROGENS AND ESTROGENS-LIKE COMPOUNDS AND COVID-19 TREATMENT

In different models of non-COVID-19 diseases, it has been shown that the administration of other molecules like conjugated estrogens and herbal compounds with estrogenic activity such *Herba epimedii*, *Semen plantaginis*, QingYan formula, phytoestrogens during four weeks in a spontaneously hypertensive rat model, decreased blood pressure, and aortic intima media thickness. Moreover, this treatment significantly increased plasma Ang-(1-7) levels, aortic Ang-(1-7), NO, and endothelial NOS (eNOS) levels and ACE2, MasR and neuronal NOS (nNOS) protein expression [87]. Thus, suggesting that estrogens or estrogens-like compounds could improve the lung injury and coagulopathy in COVID-19 severe patients. In other studies, it has been proposed that the use of clomiphene and raloxifene, a selective estrogen receptor modulator, could be employed as a COVID-19 treatment for its antiviral effects [88, 89]. In a VERO E6 cells infected with SARS-CoV-2, threated with 17β-estradiol during 24 h a reduction in viral load and reduction of TMPRSS2 levels were observed, which could impact SARS-CoV-2 entry to the cell [90]. In a randomized controlled trial, the effect of supplementation with estradiol in symptomatic post-menopausal non-severe COVID-19 females was shown, 40 patients received estradiol valerate (2 mg) orally for 7 days. In comparison with the control group a significant minor rate of reverse transcriptase-polymerase chain reaction negativization at days 5 and 7 was observed, plus a significant decrease in inflammatory markers like D-dimer, lactate dehydrogenase, and C-reactive protein was present on day 5. The authors concluded that oral estradiol can be a viable option for the management of COVID-19 [91]. This suggests that estrogens or estrogens-like compounds could improve lung injury and coagulopathy in COVID-19 severe patients. Moreover, 8 clinical studies are registered where the role of estrogens and COVID-19 is being analyzed. Table **2** summarizes the studies [92-97].

## 8 ESTROGENIC COMPOUNDS AS POTENTIAL ADJUVANT TREATMENT FOR NEUROINFLAMMATION IN COVID-19

Neuroinflammation includes the response to a stimulus by neurons, microglia, and astrocytes, and is mediated by cytokines, chemokines, and free radicals (Fig. **3A**) [98-100]. Apart from regulating the activity of neuronal circuits that control reproductive physiology, and processing of visual and auditory information, among others, estradiol activates homeostasis in neurons, astrocytes, and microglia to maintain neuronal function. This function is performed through signaling events at two types of levels, nuclear and extranuclear. The nuclear level involves classical estrogen receptors, ERα and ERβ, expressed on neurons, astrocytes, and microglia [101]. At the extranuclear level, Estrogen receptors (ERs) are in the plasma membrane, G-protein-coupled ER (GPER), or in the mitochondria (Fig. **3B**) [102, 103]. The activation of nuclear or extranuclear signaling causes the upregulation of protective molecules, such as neuroglobin [104]. In turn, neuroglobin reduces the size of lesions in the traumatized brain, inhibits apoptosis in neuronal cells, and promotes regeneration of CNS axons [105-107]. Derived from estradiol's roles in neuroinflammation, different estrogen-derived agonists or ERs-ligands have been designed for various diseases, excluding COVID-19, which involve neuroinflammation. These estrogen derivatives could be used as repurposing drugs for the adjuvant treatment of neuroinflammation in patients with COVID-19. Patent US2021008002, describes the neuroprotective effect of the compound 4-[4,4-Difluoro-1-(2-fluorophenyl) cyclohexyl]phenol, a ligand for ERβ, in a murine model of experimental autoimmune encephalomyelitis (EAE). Treatment with 4-[4,4-Difluoro-1-(2-fluorophenyl) cyclohexyl]phenol protects against whole brain, cortical and cerebellar atrophy, loss of cerebral and cerebellar neurons and synapses in gray matter, axonal loss, axonal damage, and myelin loss in spinal cord white matter [108]. Patent US2021340155 describes substituted (4-hydroxyphenyl)cycloalkane and (4-hydroxyphenyl)cycloalkene compounds as selective agonists of the estrogen receptor beta isoform and its application in the treatment of neurological, psychiatric, and/or cell proliferative diseases. In particular, compound 4-hydroxymethyl-(4′hydroxyphenyl)-cyclohexane enhances multiple types of memory dependent on the hippocampus, a brain region involved in numerous disorders, including Alzheimer's disease, depression, and schizophrenia [109]. Patent US2021230121 4-hydroxyphenyl-2H-indazol-5-ol compounds, including 2-(2-bromo-4-hydroxyphenyl)-3-chloro-2H-indazol-5-ol, 3-chloro-2-(2-chloro-4-hydroxyphenyl)-2H-indazol-5-ol, 3-chloro-2-(4-hydroxy-2-methylphenyl)-2H-indazol-5-ol, 3-chloro-2-(4-hydroxy-2-iodophenyl)-2H-indazol-5-ol, 3-chloro-2-(2-fluoro-4-hydroxyphenyl)-2H-indazol-5-ol, these compounds (ERβ ligands) increased oligodendrocyte survival, differentiation, and remyelination in murine models of EAE [110].

Patent US2021213033 describes a clinical trial wherein 82 patients received copaxone (drug indicated for the treatment of relapsing forms of multiple sclerosis) plus estriol for 24 months. The tests to determine the effect of estriol were the expanded disability status scale, 25-foot walk test, and paced auditory serial addition test. This treatment stops and reverses gray matter atrophy and disability progression in multiple sclerosis [111, 112]. The treatment of experimental autoimmune encephalomyelitis with ERβ ligand is described in patent US2021196667. Treatment (10.8 mg/kg) for 12 days provokes an increase in EAE clinical score for mice and has protective effects on EAE disease progression [113]. Patent US2015051178 describes a clinical trial in women with multiple sclerosis. This clinical trial included six-month treatment period with oral estriol (8 milligrams/day) in twelve female patients with clinically definite MS. Estriol treatment caused a significant increase in PASAT cognitive test scores. In a mouse model of EAE, estradiol reduces inflammation and demyelination, and reduces microglial/monocyte activation in white and gray matter [114]. Patent WO2014125121 proposes selective ERβ agonists, 4-(l-phenylcyclohexyl) phenol, 4-(4,4-difluoro-l-(2-fluorophenyl)cyclohexyl)phenol, l-(4,4-difluoro-l-(4-methoxyphenyl)cyclohexyl)-2-fluorobenzene and 4-(l-phenylcyclohexyl) phenol. These ERα agonists, in combination with a selective estrogen receptor β agonist, improved Morris water maze, decreased anxiety and increased levels of the amyloid β degrading enzymes, neprilysin and insulin degrading enzyme, in triple transgenic AD mice [115]. Patent US8552057, a formulation based on 17β-estradiol, genistein, daidzein increased efficacy in sustaining neuronal survival when challenged with neurotoxins, promoting expression of proteins as key players in neuroprotection and metabolism/clearance of β-amyloid in neurons/brain, and enhancing brain mitochondrial functions [116]. Finally, patent WO2008033894 describes different selective ER-β agonists, including 4-(l-phenylcyclohexyl) phenol. This compound was effective over a wide dose range (0.5 mg/kg - 10 mg/kg) in rodent models of neuropathic pain, such as mouse allodvnia studies [117]. Due to their action as ER agonists, the mentioned compounds may have the potential to be considered repurposing drugs in the treatment of neuroinflammation in COVID-19 due to their mechanism of action in neuroinflammation due to other etiologies.

## CONCLUSION

In conclusion, estrogen-containing compounds capable of increasing ACE2/Ang-(1-7)/MasR may be a therapeutic option for the management of critically COVID-19 patients, but observational studies and randomized controlled trials are needed. Also, the understanding of the SARS-CoV-2 receptor paradox is critical in order to finding an accessible solution for successfully managing COVID-19.

## Figures and Tables

**Table 1:** Neurological complications of COVID-19.

**Author**	**Complication**	**Outcomes**
Collantes, *et al*., 2021 [11]	Ischemic stroke, acute cerebral infarction, cerebrovascular disease, acute intracerebral hemorrhage, cerebral sinus venous thrombosis, encephalopathy, acute necrotizing hemorrhagic encephalopathy, oculomotor nerve palsy, isolated sudden-onset anosmia, Miller-Fisher syndrome, Guillain-Barré syndrome.	The most common neurological complication associated with SARS-CoV-2 infection was cerebrovascular disease.
Wei, *et al*., 2020 [14]	Oculomotor nerve palsy.	The virus SARS-CoV-2 infection may directly damage the myelin sheaths and surrounding axons due to complications such as oculomotor nerve palsy.
Chen, *et al*., 2021 [16]	Massive cerebral hemorrhage, acute cerebrovascular events, acute ischemic stroke, cerebral venous sinus thrombosis, generalized seizures, Meningitis/encephalitis, Guillain-Barré syndrome, Miller Fisher syndrome, and polyneuritis cranialis.	Patients, who developed acute cerebrovascular events were significantly older, more likely to present with severe COVID-19 and also more likely to present with cardiovascular risk factors, including hypertension), diabetes and previous medical history of cardiocerebrovascular diseases.
Li, *et al*., 2020 [17]	Acute intracerebral hemorrhage, cerebral sinus venous thrombosis, ischemic stroke.	Elder patients with COVID-19 may be more likely to develop acute cerebrovascular disease and more attention should be paid to older patients with cerebrovascular risk factors.
Beghi, *et al*., 2020 [18]	Guillain-Barré syndrome, encephalopathy.	People with neurological disorders might be at risk of incurring the most severe complications of the infectious disease, and, conversely, the infection itself might be a source of neurological complications.

**Table 2:** Active clinic assays where the role of estrogen in COVID-19 is analyzed.

**Drug**	**Protocol**	**Study Description**
Estrogen therapy: Norelgestromin/ Ethinyl estradiol [92]	EVRA skin patches: Norelgestromin 6 mg/Ethinyl estradiol 0.6 mg (1 patch will be placed every week for 21 days)	Parallel assignment randomized clinical trial N=60 Outcome measures: -Clinical improve to estrogen therapy in non-severe COVID-19 patients (hospitalization days, oxygen therapy, intubation or mechanical ventilation and mortality. -Symptomatic improve to estrogen therapy in non-severe COVID-19 patients (dyspnea, arthralgia, myalgia, odynophagia / pharyngeal burning, rhinorrhea, conjunctivitis or chest pain). -Biochemical improve to estrogen therapy in non-severe COVID-19 patients (Percentage change from hemoglobin, hematocrit, leukocytes, erythrocytes, platelets, prothrombin, partial thromboplastin activation time, anti-thrombin activity, fibrinogen, fibrin degradation products, D-Dimer, ALT, AST, ALP, GGT, LD, albumin, cholesterol, triglycerides, HDL, LDL, C-reactive protein, estrogens and progesterone levels, pro inflammatory cytokine and nitric oxide profile). Location of the study: Mexico Status: In recruiting
Estradiol [93]	Estradiol 100 micrograms/day for 7 days through a patch applied on the skin	Parallel assignment randomized clinical trial Outcome measures: Rate of hospitalization, Rate of Transfer to ICU, Rate of Intubation, Rate of Death for 30 days. Location: United States Status: terminated
17ß-estradiol [94]	transdermal 17ß-estradiol gel (3 mg) for ten days	Parallel assignment randomized clinical trial N=2000 Outcome measures: -Evidence of disease progression for mild cases and in hospitalized patients for 28 days. -Mortality, duration in hospital, admission to ICU, need for renal replacement therapy, ventilation and time of discharge during 28 days. Location of the study: Qatar Status: not yet recruiting
Estradiol cypionate and progesterone [95]	Estradiol Cypionate 5mg intramuscular injection at admission and Progesterone 200 mg by mouth daily for 5 days starting at admission.	Parallel assignment randomized, placebo controlled clinical trial. N=20 Outcome measures: -The proportion of patients who achieve scores 1 or 2 on the 9-point World Health Organization (WHO) ordinal scale through day 28. - Length of hospital stay, readmission, duration of mechanical ventilation, time and cause of death, change in biological markers ferritin, procalcitonin and troponin, C-reactive protein, D-Dimer, fibrinogen, ALT, AST and LDH, neutrophil:lymphocyte ratio, grade 3 and 4 adverse events occurrence and serious adverse events occurrence (time frame Day 14, Day 28, Day 60). Location to the study: United States Status: Recruiting
Estetrol monohydrate (E4) [96]	One Estetrol monohydrate (E4) 15 mg tablet once per day daily for 21 consecutive days	Parallel assignment randomized clinical trial N=162 Outcome measures: -Percentage of participants who have recovered at day 28. -Time to recovery, SARS-CoV-2 viral load and number of participants with adverse events monitoring for 28 days. Location: Belgium, Hungary, Poland and Russian Federation. Status: active, not recruiting
Raloxifene [97]	Raloxifene was administered as 60 mg hard gelatin capsule(s) once a day. Starting from day 2 of treatment: one single capsule (plus one of placebo to guarantee the blinding) containing 60 mg raloxifene was administered in Group 1, and 2 capsules 60 mg each for a total of 120 mg in Group 2. The treatment was taken by the patients for two weeks.	Parallel assignment randomized, placebo controlled clinical trial N=68 Outcome measures: -Proportion of participants with undetectable SARS-CoV-2 at PCR randomization (time frame: Day 7). -Proportion of participants not requiring oxygen therapy and/or mechanical ventilation (time frame: Day 14). -Proportion of participants with undetectable SARS-CoV-2 at PCR (Time Frame: At days 14 and 28). -Proportion of participants not requiring oxygen therapy and/or mechanical ventilation (Time Frame: At days 7 and 28]. -Proportion of patients in each National Early Warning Score (NEWS) category (Time Frame: At days 7, 14, 28). -Mean value of National Early Warning Score (NEWS) category (Time Frame: At days 7, 14, 28). -Proportion of participants with any adverse event with grade ≤ 2 after randomization (Time Frame: At days 7, 14, 28). -Proportion of participants with any severe adverse events (grade ≥ 3 according to CTCAE) after randomization (Time Frame: At days 7, 14, 28). -Proportion of hospitalized participants who at the beginning of the study were in domicile isolation after randomization. (Time Frame: At days 7, 14, 28). -Proportion of participants admitted to intensive care after randomization. (Time Frame: At days 7, 14, 28). -Proportion of survivors (Time Frame: At days 7, 14, 28). -Mean change from baseline to day 7, 14, 21 and 28 after randomization of value for biomarker parameters (Time Frame: At days 7, 14, 21 and 28). -Quality of life questionnaire (Time Frame: At month 3). Locations to the study: France, Italy and Spain. Status: Completed

## LIST OF ABBREVIATIONS

ACE: Angiotensin-converting Enzyme
ADAM17: A Disintegrin and Metalloproteinase 17
Ang-(1-7): Angiotensin-(1-7)
AngII: Angiotensin II
AT1R: Angiotensin II Receptor Type 1
AT2R: Angiotensin II Receptor Type 2
BBB: Blood-brain Barrier
BDNF: Brain-derived Neurotrophic Factor
CCL2: C-C motif Chemokine Ligand 2
CHC: Hormonal Contraception
CI: Confidence Interval
CNS: Central Nervous System
COVID-19: Coronavirus Disease 2019
DM2: Diabetes Mellitus Type 2
EAE: Autoimmune Encephalomyelitis
eNOS: Endothelial Nitric Oxide Synthase
ERK: Extracellular Signal-regulated Kinase
ERs: Estrogen Receptors
GABA: Gamma-aminobutyric Acid
GPER: G-protein-coupled Estrogen Receptor
GTEx: Genotype-Tissue Expression
HPA: Hypothalamus-pituitary Adrenal
hsCRP: High-sensitivity C-reactive Protein
IL: Interleukin
JNK: c-Jun N-terminal Kinase
LDH: Lactate Dehydrogenase
LMWH: Low Molecular Weight Heparin
LPS: Lipopolysaccharide
MAPK: Mitogen-activated Protein Kinase
MasR: Mas Receptor
MCAS: Mast Cell Activation Syndrome
MHT: Menopausal Hormone Therapy
Mn-SOD: Manganese Superoxide Dismutase
NADPH: Reduced Nicotinamide Adenine Dinucleotides Phosphates
NF-κB: Nuclear Factor Kappa-light-chain-enhancer of Activated B Cells
NMDA: N-methyl-D-aspartate
nNOS: Neuronal Nitric Oxide Synthase
NO: Nitric Oxide
NOS: Nitric Oxide Synthase
OR: Odds Ratio
PI3K: Fosfatidilinositol-3-kinasa
PKC: Protein Kinase C
PLC: Phospholipase C
PNS: Peripheral Nerve System
RAS: Renin-angiotensin System
ROS: Reactive Oxygen Species
SARS-CoV-2: Severe Acute Respiratory Syndrome Coronavirus 2
TGF: Transforming Growth Factor
TMPRSS2: Transmembrane Serine Protease 2
TNF-α: Transforming Growth Factor α
